# New Insights into Ligand-Receptor Pairing and Coevolution of Relaxin Family Peptides and Their Receptors in Teleosts

**DOI:** 10.1155/2012/310278

**Published:** 2012-09-13

**Authors:** Sara Good, Sergey Yegorov, Joran Martijn, Jens Franck, Jan Bogerd

**Affiliations:** ^1^Department of Biology, University of Winnipeg, Winnipeg, MB, Canada R3B 2E9; ^2^Department of Biology, Faculty of Science, University of Utrecht, 3584 CH Utrecht, The Netherlands

## Abstract

Relaxin-like peptides (RLN/INSL) play diverse roles in reproductive and neuroendocrine processes in placental mammals and are functionally associated with two distinct types of receptors (RXFP) for each respective function. The diversification of RLN/INSL and RXFP gene families in vertebrates was predominantly driven by whole genome duplications (2R and 3R). Teleosts preferentially retained duplicates of genes putatively involved in neuroendocrine regulation, harboring a total of 10-11 receptors and 6 ligand genes, while most mammals have equal numbers of ligands and receptors. To date, the ligand-receptor relationships of teleost Rln/Insl peptides and their receptors have largely remained unexplored. Here, we use selection analyses based on sequence data from 5 teleosts and qPCR expression data from zebrafish to explore possible ligand-receptor pairings in teleosts. We find support for the hypothesis that, with the exception of RLN, which has undergone strong positive selection in mammalian lineages, the ligand and receptor genes shared between mammals and teleosts appear to have similar pairings. On the other hand, the teleost-specific receptors show evidence of subfunctionalization. Overall, this study underscores the complexity of RLN/INSL and RXFP ligand-receptor interactions in teleosts and establishes theoretical background for further experimental work in nonmammals.

## 1. Introduction

Relaxin-like peptides are members of the insulin superfamily and, like insulin and insulin-like growth factors (IGF), are small peptides (~60 amino acids) that share a common two-domain structure (A and B domains) in their mature form [1]. Functionally, however, relaxin family peptides are different from insulin and IGF: they bind to unrelated receptors and play diverse roles in reproduction and neuroendocrine regulation as opposed to carbohydrate/fat metabolism and growth. Four relaxin family peptide-encoding genes (*RLN*, *RLN3, INSL3*, and *INSL5*) originated early in vertebrate history and are shared by most vertebrates [2]. The receptors for the RLN/INSL peptides belong to two distinct groups of G protein-coupled receptors (GPCR), collectively named the relaxin family peptide receptors (RXFP) [3].

In mammals, there are four known receptors, RXFP1–4, associated with the four relaxin family ligands. RXFP1 and RXFP2 are evolutionarily related to glycoprotein hormone receptors (e.g., luteinizing and follicle-stimulating hormone receptors), containing a large extracellular domain made up of ten leucine-rich repeats (LRR) and a low-density lipoprotein receptor type A (LDLa) module; they are the cognate receptors for the ligands RLN and INSL3 in humans, both of which primarily have reproductive actions [3]. On the other hand, RXFP3 and RXFP4 are classic type I peptide GPCRs with short N-terminal domains; they are evolutionarily related to somatostatin and angiotensin receptors and, in humans, are the cognate receptors for RLN3 and INSL5, both of which are associated with neuroendocrine signaling [3].

The two hormones with reproductive functions in mammals, RLN and INSL3, are the best understood. The hormone RLN is well known for its role in parturition, where it softens connective tissues of the reproductive tract via tissue remodeling and prepares the mammary glands for lactation, but it has numerous other physiological actions as well [1]; its receptor (RXFP1) also exhibits a wide distribution suggesting endocrine action in mammals [7] (Table S1, see supplementary materials available online at doi: 10.1155/2012/310278). In teleosts, the peptide sequence of Rln is highly similar to that of Rln3 [8]; although its function remains unknown, the *rln* gene exhibits substantial overlap in expression with* rln3*, both being highly expressed in brain, although teleost *rln* is also significantly expressed in gonads [9]. While mammalian and teleost *RLNs* differ somewhat in their expression patterns, *INSL3* has a more similar expression pattern in the two lineages; it is highly expressed in Leydig cells in both mammals [10] and teleosts [8], and at lower levels in other tissues (see Table S1). In mammals, the receptor for INSL3, RXFP2, is also highly expressed in testes suggesting paracrine action [6], but lower levels of RXFP2 expression are observed in a wide array of tissues [7]. The receptor has been, until now, unstudied in teleosts.

The peptides RLN3 and INSL5 exert their influence primarily through the hypothalamic-pituitary-gonadal (HPG) axis [11, 12]. RLN3 is the most conserved member of the family; it is predominantly expressed in the nucleus incertus (NI) in mammalian brain [13] and its homologous region in teleosts [14]. Ascending RLN3-producing projections from the NI innervate a broad range of RXFP3-expressing regions of the forebrain in mammals, including the hypothalamus and it is implicated in the acute stress response and regulation of food intake [12, 15]. Collectively, these lines of evidence suggest that RLN3 acts through the HPG axis and may play a dual role linking nutritional status to reproductive function [12]. Lastly, INSL5 is the least well understood member of the family, but in humans its primary sites of expression are rectum, colon, and uterus [16, 17] (see Table S1). The receptor for INSL5 in mammals, RXFP4, has a wide distribution being found in colon, placenta, testis, thymus, prostate, kidney, and brain in human [18], strongly suggesting endocrine action.

Despite the evolutionary distance separating RXFP1/2 and RXFP3/4-type receptors, experimental studies have shown that some RLN/INSL peptides can bind additional (secondary) receptors at lower affinity [5]. For example, in addition to RXFP3, RLN3 can bind to and activate RXFP1 and RXFP4, RLN can bind to RXFP2 in addition to RXFP1 [19], and INSL5 can bind to (but activate only weakly) RXFP3 in addition to its primary receptor RXFP4. Such “primary” and “secondary” ligand-receptor interactions have been demonstrated for human RLN/INSL-RXFP pairs, but analogous pairings in other vertebrates, such as teleosts, in which relaxin family peptide-receptor signaling and diversification have taken an evolutionary pathway distinct from that in mammals [2], remain to be established.

**Figure 1:**
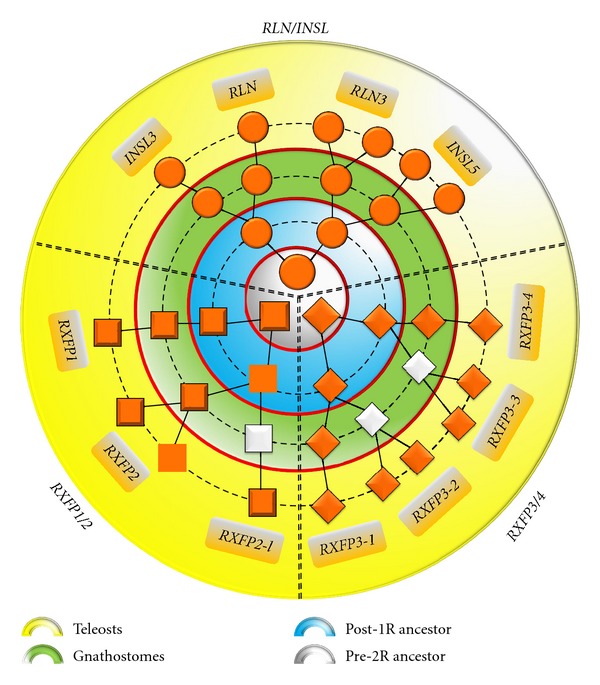
The role of Whole Genome Duplications (WGD) in the expansion of the *RLN/INSL* and *RXFP* genes in vertebrates. The three gene families (one ligand (circles) and two receptor (squares) families) arose as a result of WGDs (1R, 2R, and 3R) from three ancestral genes. *RLN/INSL* peptides: following 1R, there were two *RLN/INSL*-like genes, following 2R one of these gave rise to *RLN3* and *INSL5*, and the other to *RLN* and *INSL3*. After the teleost fish-specific WGD (3R), the duplicates of *rln3* and *insl5* were retained bringing the total number of *rln*/*insl* genes in teleosts to 6. *RXFP3/4* receptors: four *RXFP3/4*-type receptor genes were generated from a single-ancestral gene during 2R; all four of these genes were retained in teleosts, but in tetrapods, only two receptors, *RXFP3* (termed *RXFP3-1*) and *RXFP4* (*RXFP3-4*), were retained. After 3R, the duplicates of *rxfp3-2* and *rxfp3-3 *were retained. *RXFP1/2* receptors: most vertebrates have only a single copy of *RXFP1* and *RXFP2,* a few (opossum, frog, reptiles, and zebrafish) have *RXFP2-like*. Layers coloured in four distinct colors indicate ancestral stages (legend below); WGDs are depicted as red lines surrounding these ancestral stages. White shapes indicate genes lost in most (*RXFP2-l*, *RXFP3-3*) or all (*RXFP3-2*) tetrapod lineages. *RXFP2-l= RXFP2-like*. Based on Yegorov and Good [2].

**Figure 2:**
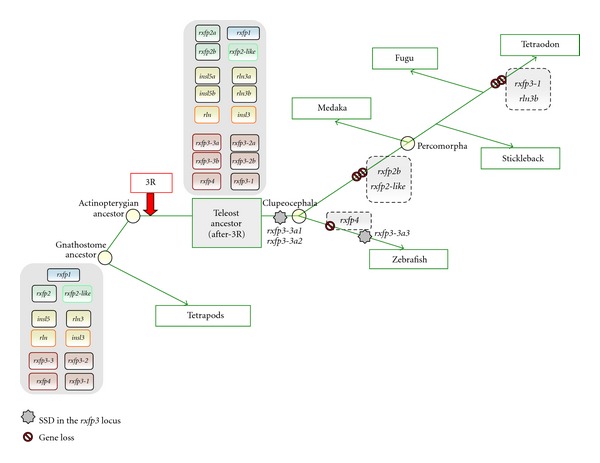
Post 3R gene loss and gain in five teleost fish species. Following 3R, teleosts start with a gene set composed of 10 receptors and 6 ligands. Prior to divergence of zebrafish, *rxfp3-3a* is locally duplicated, generating tandem genes *rxfp3-3a1* and *rxfp3-3a2*. Zebrafish retains most of the genes, except *rxfp4*, but gains an additional copy of the *rxfp3-3* gene, *rxfp3-3a3*, through SSD. Other teleosts lose *rxfp2-like* and also the 3R-duplicate *rxfp2b*. SSD: small-scale (local) duplication. 3R: fish-specific WGD. Data from Yegorov and Good [2]. Phylogeny and classification of fish adapted from Kinoshita et al. [4].

**Figure 3:**
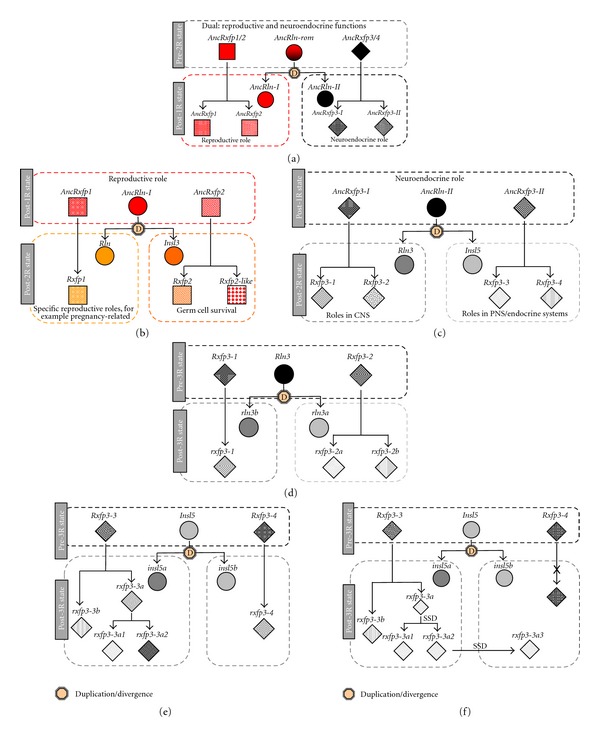
The hypothesized functional diversification of the *rln*/*insl* and *rxfp* genes in the gnathostome ancestors (a, b, and c) and in teleosts (d, e, and f). (a) The pre-1R three-gene system gave rise to two ligand genes and two pairs of receptor genes following 1R. After 1R, both ligands and receptors are structurally and functionally identical, which is favorable for promiscuous ligand-receptor interactions, in combination with selective pressures promoting a division of reproductive and neuroendocrine systems, leading to the establishment of novel ligand-receptor pairs. (b) Duplication and divergence of the *rln-rxfp1* and *insl3-rxfp2* ancestor genes. On the basis of the proposed relatedness of *rxfp2-like* to *rxfp2*, we hypothesize that Rxfp-like, at least immediately after 2R, functioned as a receptor for Insl3. (c) Duplication and divergence of the genes ancestral to *rln3* and *insl5* and their rxfp3/4-type receptor genes. Since all tetrapods lost *rxfp3-2* and most of them also lost *rxfp3-3*, their ligand-receptor pairs lost their ancestral three-component nature and became two-component, that is, Rln3*-*Rxfp3-1 and Insl5*-*Rxfp4. (d) Teleosts retained all after-2R rxfp3/4 receptor genes and seem to have experienced further subfunctionalization with the formation of complex ligand-receptor relationships. We hypothesize a functional specialization of the two *rln3* paralogs to work with *rxfp3-1* (*rln3a*) and two *rxfp3-2* genes. (e) Diversification of *rxfp3-3* and *rxfp3-4* genes in percomorpha (f) Zebrafish has lost its *rxfp3-4* (i.e., *rxfp4*) gene but has an extra copy of *rxfp3-3a3*, which may imply that the receptor of Insl5b is Rxfp3-3a3. Note that in (b) and (c) *insl5* paralogs are chosen arbitrarily and the interaction of the peptide with the receptors can be reversed; that is, Insl5a may function with Rxfp3-4 and Insl5b may interact with Rxfp3-3 receptors SSD = small scale duplication.

Recent evolutionary analyses revealed that vertebrate *RLN/INSL* genes and their receptors primarily diversified through the two rounds (2R) of whole genome duplication (WGD) that occurred in early vertebrate evolution and, in teleosts, during the teleost fish-specific WGD (3R) (Figure 1). To summarize, mammals retained 4 ligand and 4 receptor genes following 2R, while teleosts have 10 (most teleosts) or 11 (zebrafish) receptor and 6 ligand genes following 3R (Figure 1) and after-3R local duplications (Figure 2, Table S2). Many of the genes retained in duplicate in fish (*rln3-*, *insl5-*, and *rxfp3*-type genes) are hypothetically involved in neuroendocrine regulation (Figure 3). But due to a lack of understanding of the evolutionary history of *rln/insl* and *rxfp* genes in teleosts, the ligand-receptor pairings in teleosts are virtually unknown.

**Figure 4:**
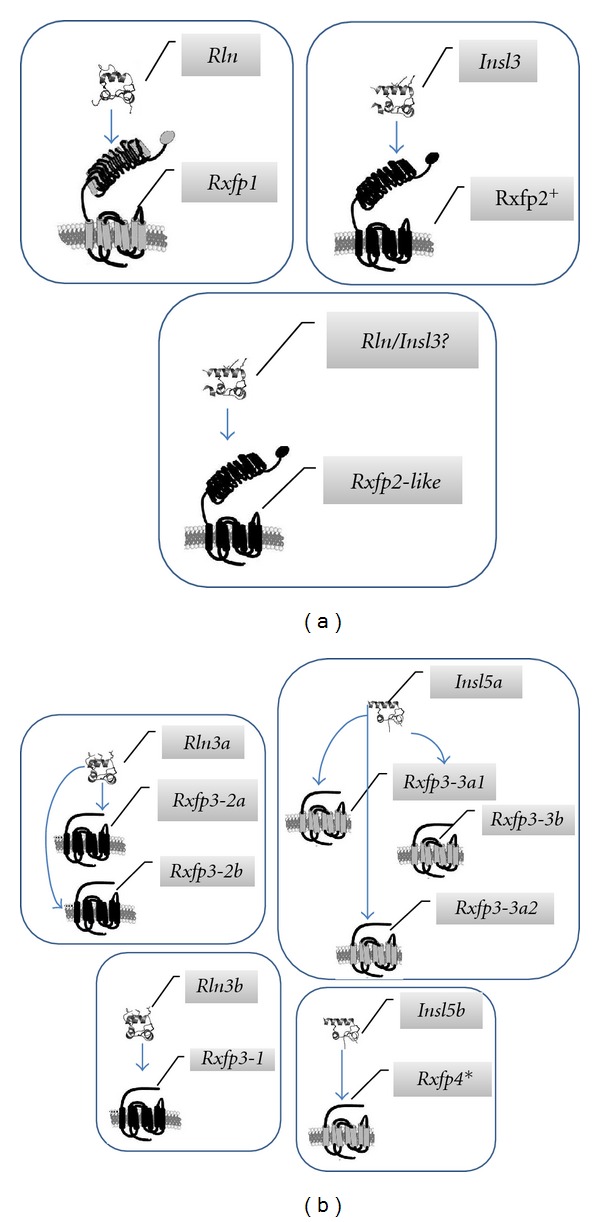
Ligand-receptor pairings of the Rln/Insl peptides and their Rxfp receptors putatively associated with (a) reproductive and (b) neuroendocrine processes in teleosts as hypothesized based on mammalian pairings and on their gene duplication history (see Figures 1 and 3). Few tetrapods (reptiles, frog, and opposum) and zebrafish have been found to possess the receptor *rxfp2-like*, which is phylogenetically more closely related to *rxfp2 *than *rxfp1*, but still of ancient origin [2]. However, the lack of *insl3* in the reptiles, that also harbour *rxfp2-like* (data from [2]), suggests that Rln may be an alternate ligand. ^+^Zebrafish retained two 3R paralogs of *rxfp2*, *rxfp2a*, and *rxfp 2b*, while the remaining teleosts appear to have lost one copy. *In zebrafish, the *rxfp4* gene was lost and possibly replaced by *rxfp3-3a3* (see Figures 3 and 4). Images of receptors and peptides adopted with permission from the publisher for Halls et al. [5] and Kong et al. [6].

One of the interesting aspects of the evolution of RLN/INSL peptides is how a set of relatively closely related ligands signals via two unrelated types of receptors. Yegorov and Good [2] hypothesized that this dual-functioning arose in the ancestral pre-2R RLN/INSL peptide that had roles in both reproductive (via RXFP1/2-receptor) and neuroendocrine (via RXFP3/4) regulations in primitive vertebrates (Figure 3). As a result of the WGDs, the ancestral tripartite system gave rise to two distinct parties of RLN/INSL-RXFP ligand-receptor pairs (Figure 3). Curiously, it can be observed that, with the exception of the RXFP1 receptor and its ligand RLN, each of the duplication events resulted in a single ligand that potentially could function with two related receptors (Figure 3). In most mammals, this tripartite model became reduced to a 1 : 1 relationship for ligands and receptors after the divergence of tetrapods from the gnathostome ancestor (as described above), but in teleosts, there are multiple receptors for some ligands, which may have occurred through receptor subfunctionalization (Figure 3). Based on the evolutionary history of duplication, and the ligand-receptor pairings in mammals, we developed hypotheses concerning which ligand-receptor pairings we expect in teleosts (Figure 4). The primary goal of this paper is to test our hypotheses about the Rln/Insl-Rxfp ligand-receptor pairs in teleosts using selection analyses and experimental qPCR data from zebrafish.

## 2. Results

### 2.1. Selection Analyses

We performed two kinds of molecular evolutionary analyses to (1) hypothesize which ligand-receptor pairings may occur in teleosts and (2) examine differences in selection among mammalian and teleost genes.

 (1) Previous studies have used the correlation of evolutionary distances between putative ligand-receptor pairs as evidence of cofunctioning [20, 21]. Here, we employed a similar correlation approach, but rather than comparing the mean evolutionary distances among gene pairs, we compared the proportion of sites under different forms of selection (purifying, neutral, or positive) in pairs of teleost genes to the “primary” ligand-receptor pairs known to exist in mammals, *rln*-*rxfp1*, *insl3*-*rxfp2*, and *rln3*-*rxfp3-1.* If the genes coding for the ligands and receptors coevolve, we expect a correlation in the rates and types of selection on ligand-receptor pairs. This would correspond to values falling along the (0, 0 : 1, 1) plane of the XY-plot. On the other hand, a similar [X,Y]-value for the same ligand-receptor pair in mammals and teleosts would suggest that the pair plays a similar role in the two lineages.

(2) We tested for evidence of (a) codon-specific positive selection in mammalian and teleost ligand and receptor genes and (b) codon-specific positive selection in mammalian versus teleost genes using the branch-site model of positive selection. While the first analysis (a) tests whether specific codons have been positively selected within lineages, the second (b) looks for evidence that codons have been differentially selected in mammalian versus teleost lineages.

**(1) Evidence for Ligand-Receptor Coevolution for Mammalian and Teleost Orthologs**

**Figure 5:**
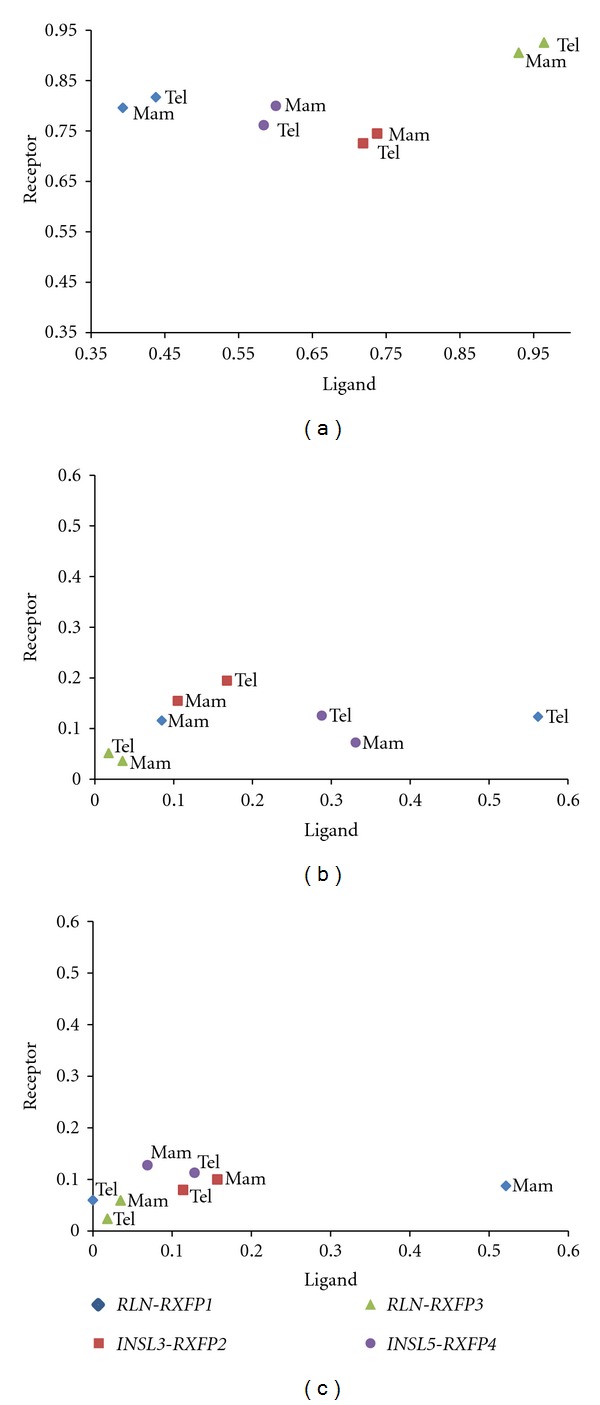
Estimated proportion of sites in the ligand (*x*-axis) and receptor (*y*-axis) genes evolving under (a) purifying, (b) neutral, and (c) positive selection in the genes of the putative ligand-receptor pairs of the RLN/INSL-RXFP system in mammals and teleosts.

Between 70 and 93% of the sites across all genes, and in both mammals and teleosts, have been subject to purifying selection (Figure 5(a)). Additionally, the extent of purifying selection was symmetric for the ligand-receptor pairs *rln3-rxfp3* and *insl3-rxfp2* suggesting close coevolution, while for the remaining two pairs, *rln-rxfp1* and *insl5-rxfp4*, the proportion of sites under purifying selection was higher for the receptor genes (between 0.7 and 0.92) than for the ligands (ranging from 0.4–0.95), suggesting a more diffuse coevolution (or no coevolution), and more relaxed evolution on the ligand.

On the other hand, there are significantly fewer sites which are evolving neutrally (Figure 5(b)) or are subject to positive selection (Figure 5(c)). For the receptor genes, from 3 to 20% of the sites were found to be evolving neutrally (Figure 5(b)), and from 2 to 13% were subject to positive selection; *rxfp3* exhibits the fewest neutral or positively selected sites, *rxfp4* has the highest proportion of sites under neutral evolution and *rxfp2* exhibits the highest proportion of sites under positive selection. Largely due to the anomalous nature of asymmetric selection on the *rln-rxfp1* ligand-receptor system in mammals, the extent of neutral and positive selection among ligand genes varied widely between mammals and teleosts, primarily because teleost *rln *was found to have a large number of sites evolving neutrally, whereas mammalian *RLN* has a large proportion of sites subject to positive selection (Figures 5(b) and 5(c), resp.).

**The Selection Analysis Supports Our Hypothesis for Many Ligand-Receptor Pairs in Teleosts, but the Receptors for the Two Insl5 Paralogs Remain Unclear**

**Figure 6:**
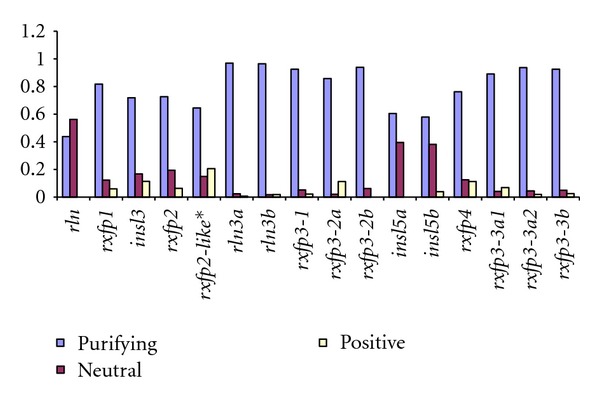
The proportion of sites in ligand and receptor genes subjected to different kinds of selection in teleosts. Selection types: purifying (light purple), neutral (dark purple), and positive (yellow). Hypothesized ligand-receptor pairs are placed in consecutive order. *Only present in zebrafish.

Given the presence of additional ligand and receptor genes in teleosts for which no ortholog was present in mammals, the correlation approach could not be used for the additional ligand-receptor genes in teleosts because there was no reference comparison in mammals and too many possible pairs to consider. Thus, to examine the possible pairings of these additional genes, we simply plotted the proportion of sites subject to each form of selection in teleosts for visual comparison (Figure 6). This revealed that the gene coding for Rln has a higher number of neutrally evolving sites than the gene of its proposed receptor, Rxfp1, although this may be an artifact of the comparison to mammalian RLN. On the other hand, the numbers of selected sites in the genes of the proposed ligand-receptor pairs *insl3-rxfp2 *(as demonstrated above)*, rln3a-rxfp3-2a/rxfp3-2b,* and *rln3b-rxfp3-1* were similar, supporting possible cofunctioning, although *rxfp3-2a* shows a higher fraction of positively selected sites than either of the *rln3* ligand genes. Lastly, however, there was also a poor correlation in the expected selection profile of *insl5* compared with its proposed receptor genes: both teleost *insl5a* and *insl5b* evolve relatively neutrally but none of their proposed receptors do, with the exception of *rxfp4*, which has a slightly higher rate of neutral and positive selection. The remaining three *rxfp3-3* receptor genes are very conserved (Figure 6). Thus, although teleost *insl5* and *rxfp4* genes had similar selection profiles to those of mammals (see above), suggesting a conserved function between the two lineages, the other three proposed receptors for the *insl5* paralogs (i.e., *rxfp3-3a1, rxfp3-3a2* and *rxfp3-3b*) exhibited strong purifying selection and did not closely parallel the selection profile of either candidate ligands.

**(2a) Evidence for Codon-Specific Positive Selection in Mammalian and Teleost Ligand and Receptor Genes**

To look for evidence of codon-specific positive selection in mammalian and teleost lineages, we compared models 7 (purifying selection), 8 (positive selection), and 8a (relaxation of purifying selection) using maximum likelihood-based comparisons [22] in mammals and teleosts. Genes are considered to be under positive selection if the support for model 8 is greater than model 7, but also model 8a. For genes that exhibited evidence of positive selection, determination of the amino acid sites estimated to be under selection was tested using Bayesian Empirical Bayes (BEB). We found evidence of positive selection for mammalian *INSL5* and mammalian *RLN*; however, the hypothesis that the positive selection found in mammalian *INSL5* is actually caused by a relaxation of purifying selection (i.e., tested by comparing model 8a versus model 8) could not be rejected. The extent of positive selection on mammalian *RLN *is extensive; however, in total, 12 amino acid positions were identified as having a BEB probability > 0.9 that *ω* > 1.0 (i.e., to be under positive selection) and another five had a probability > 0.8 that *ω* > 1.0 (Table S3). This suggests the presence of strong diversifying selection on mammalian *RLN*. In teleosts, only *insl3* showed evidence of having codons subject to positive selection at two sites (Table S3).

There was some, but limited, evidence of positive selection on the receptor genes within mammalian or teleost lineages. Only one codon was found to exhibit strong evidence of positive selection in mammalian *RXFP1*, and two for *RXFP2*, while three codons showed evidence of positive selection in fish *rxfp2*, but the latter hypothesis was more likely attributed to a relaxation of purifying selection. Additionally, a few codons were found to have evidence of positive selection in mammalian *RXFP3* and teleost *rxfp4* (stronger evidence). Although mammalian *RXFP4* also showed evidence of positive selection (model 8 was preferred over models 7 and 8a); no specific codons had a BEB probability of being under strong positive selection. Overall, this suggests similar patterns of selection on ligand-receptor pairs, with the notable exception of *RLN-RXFP1* in mammals for which strong evidence of positive selection exists for the ligand, but no strong evidence of positive selection on the mammalian receptor gene, *RXFP1*.

**(2b) Evidence for Differential Selection on Teleost Versus Mammalian Lineages for Orthologous Receptors**

**Figure 7:**
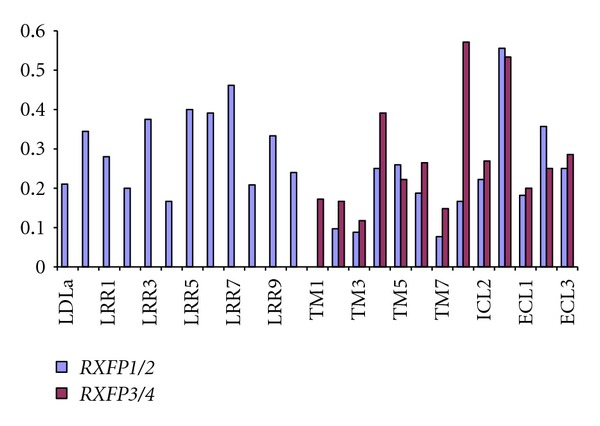
The proportion of amino acids selected per region in *RXFP1/2* and *RXFP3/4* receptor genes that showed evidence of positive selection in mammalian or teleost lineages according to the branch-site test of positive selection. LDLa—low-density lipoprotein module A, LRR—leucine rich repeat, TM—transmembrane domain, ICL—intracellular loop, and ECL—extracellular loop.

Although the above analyses suggested that only mammalian *RLN* has experienced high levels of codon-specific positive selection, using the branch-site model of codon-specific positive selection, we tested whether mammalian and teleost lineages have been subject to lineage-specific positive selection, that is whether they have been selected to be fixed for different amino acids (Table S5). This analysis revealed considerable evidence of lineage-specific selection indicating that mammalian and teleost lineages have evolved in different ways, and it also highlighted some important differences in the regions of the receptors that have been subject to positive selection. By mapping, codons were found to have evidence of positive selection to their position in the mature proteins; we find that (1) the low-density lipoprotein/leucine rich repeat (LDL/LRR) region of *RXFP1/2*-type genes is an important region of diversification among lineages; (2) for the 7 transmembrane (7TM) region shared between the two receptor types, all regions have more selected sites in *RXFP3/4-* than in *RXFP1/2-*type genes, except extracellular loop 2 (ECL2), and (3) intracellular loops 1 (ICL1) and 3 (ICL3) have many positively selected sites for *RXFP3/4* genes while ICL3 also has many amino acids selected for *RXFP1/2 *type genes (Figure 7).

Closer examination of the sites that were selected in mammalian versus teleost lineages revealed somewhat different regions of selection in teleosts versus mammals. For *RXFP1*, mammals had more selection on the first few domains of the LDLa/LRR region, while teleosts exhibit greater selection on the terminal LRR domains. Additionally, in general mammalian, *RXFP1* genes were found to have more selected sites in the ICLs (ICL1 and ICL3), while teleosts exhibit more selection in the ECLs (ECL1 and ECL3) (Figure S1). This suggests that while the overall patterns of selection are similar among mammalian and teleost putative ligand-receptor orthologs, divergent selection has operated in both lineages for all genes, and some of this selection could be associated with intra- versus extracellular signaling (Figure S1).

**Quantitative Expression of All Ligand and Receptor Genes in Zebrafish across Multiple Tissues**

**Figure 8:**
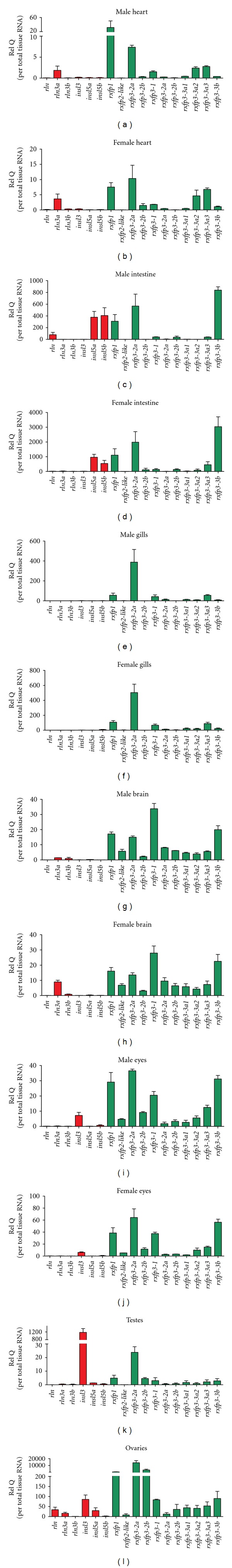
Relative expression of *rln/insl* and *rxfp* genes in zebrafish tissues. The expression of a gene relative to the average expression across all genes in a given tissue of males and females is shown. Red and green bars indicate the relative expression of the ligand and receptor genes, respectively. Three biological replicates were used to determine the standard errors on the relative expression.

To infer functional ligand-receptor relationships, we assessed the expression of both ligand and receptor genes in male and female zebrafish heart, intestine, gonads, muscle, gills, brain, and eyes using real-time, quantitative PCR. Overall, the fold increase of the target to housekeeping genes, especially the receptors, was similar for both sexes in all tissues (except gonad) confirming the reliability of the data (Figures S2 and S3). To allow comparison of the relative amounts of mRNAs produced per tissue, the relative mRNA expression levels were normalized to the total amount of RNA isolated per tissue (Figure 8). This revealed that for all tissues studied, the expression levels of all *rxfp* genes appeared to be higher than the expression levels of all *rln*/*insl* genes, except for the very high expression levels of *insl3* in testis tissue (Figure 8).

The ligand *rln* was most abundantly expressed in gonads and male intestine (Figures S2 and 8); its primary hypothesized receptor, *rxfp1, *was also highly expressed in gonads, as was a potential secondary candidate receptor, *rxfp2a* (Figure 8). The* rxfp1* transcript was also detected in male heart and brain, while *rxfp2b* expression was found in brain and eyes. Expression of the zebrafish-specific *rxfp2-like *transcript, a candidate receptor for Rln and Insl3, was only found in brain at high levels. Very high expression of *insl3* mRNA was found in testes and somewhat lower levels in ovaries and eyes. The primary candidate receptors for Insl3 are Rxfp2a and Rxfp2b, and high expression of both *rxfp2a* and *rxfp2b* was observed in gonads, while *rxfp2-like* was not detected in testes or ovaries. As expected, *rln3a* and *rln3b* expression was found predominantly in brain and gonad, but we also identified *rln3a* expression in heart (Figures S2 and 8). On the other hand, all of the *rxfp3-1*, *rxfp3-2*, and *rxfp3-3* genes showed a similar expression pattern: high expression in brain with lower levels in testes and eye, only *rxfp3-3a3* exhibited relatively low expression in brain. Relatively high levels of *insl5a* and *insl5b* mRNA were found in intestine, but additionally *insl5a* expression was found in gonads and brain. Our hypothesized candidate receptors for Insl5a are Rxfp3-3a1, Rxfp3-3a2, and Rxfp3-3b and for Insl5b is Rxfp3a3 (Figure 4): of the genes coding for these receptors, only *rxfp3-3b* showed high expression in the intestine (Figure 8).

## 3. Discussion

The main goal of this paper was to explore possible ligand-receptor pairings for the *rln*/*insl*-*rxfp* genes in teleosts. Based on previous bioinformatic analyses, we describe how teleosts preferentially retained 2R- and 3R-derived paralogs of genes putatively involved in neuroendocrine functions (*rln3*/*insl5*-*rxfp3/4*), ultimately leading to a greater number (10-11) of receptor genes than ligands (6). Given that the ligand-receptor pairings in teleosts are largely unknown, we employed selection and expression analyses to explore the possible ligand-receptor pairings. Overall, the selection analyses showed that (1) the extent of purifying, neutral, and positive selection acting on the four *RLN-RXFP* orthologs was highly similar between mammalian and teleost genes suggesting that, with the exception of mammalian *RLN*, ligands and receptors have the same binding relationships in both lineages and (2) the ligand-receptor pairs *RLN3*-*RXFP3* and *INSL3*-*RXFP2* exhibited highly similar selection profiles suggesting close coevolution, while the pair *INSL5-RXFP4* exhibited a more diffuse coevolution, and *RLN-RXFP1* exhibited much faster evolution of the ligand in mammals than in teleosts. The overall similarity between the genes in teleosts and mammals is supported by the observation that all of the teleost ligand genes exhibit predominant expression in the same tissues as their orthologs in mammals: rln and insl3—gonad, rln3—brain and insl5—intestine. However, even if the binding relationships are the same, it does not mean that the gene pairs have the same function in mammals and teleosts; indeed, the branch-site test of positive selection suggests that differentiation in function has occurred between the two groups. Secondly, although the binding relationships of the genes with orthologs in mammals and teleosts may be the same, it was difficult to resolve the ligand-receptor pairing relationships for the additional genes found in teleosts, but not in mammals. 

### 3.1. The Highly Conserved Pair RLN3-RXFP3 Expanded through Gene Duplication and Possible Subfunctionalization in Teleosts

**Figure 9:**
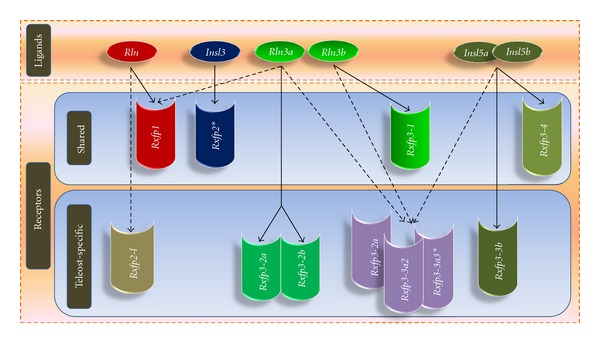
Proposed Rln/Insl-Rxfp ligand-receptor pairings based on previous genomic data (see Figure 4) and the analyses presented here. Solid lines represent potential ligand-receptor relationships that are well supported, while dashed lines represent uncertain pairings. There are four receptor orthologs between mammals and teleosts, and the data support the same ligand-receptor pairings for these genes in teleosts. For the seven teleost-specific receptors, strong ligand-receptor pairings were supported for three (solid lines), while the other three Rxfp3-3 receptors have unclear pairings (dotted lines), and Rxfp2-like is a probable secondary receptor for Rln.

The *RLN3-RXFP3* system shows strong evidence of ligand-receptor coevolution with almost all amino acids being subject to purifying selection for both genes, and exhibiting a nearly perfect correlation in both mammals and teleosts. These findings are in accordance with previous studies and further support hypotheses about the highly conserved nature of the *RLN3-RXFP3* genes, and their probable parallel function across most vertebrates [23]. However, teleosts possess two 3R-derived *rln3* paralogs (*rln3a* and *rln3b*) and multiple *rxfp3*-type genes, not all of which are orthologous to mammalian *RXFP3*. Based on the duplication history of the genes [2], we proposed that the Rln3 peptide together with Rxfp3-1 and Rxfp3-2 receptors formed a tripartite ancestral teleost ligand-receptor signaling system (Figure 3), and hypothesized that the after-3R subfunctionalization of the *rln3* paralogs would be associated with subfunctionalization of the *rxfp3-1* and *rxfp3-2* receptor genes (Figures 3 and 4). Taking into account that in *Tetraodon nigroviridis* the loss of *rln3b* coincides with the pseudogenization of *rxfp3-1* (Figure 3), we further propose that Rln3b is a cognate ligand of Rxfp3-1, while Rln3a has specialized to function with two receptors, namely, Rxfp3-2a and Rxfp3-2b (Figure 9). This hypothesis is supported by experimental data presented here and elsewhere. For example, experimental studies performed in zebrafish [14] and eel [9] indicate that the expression of the *rln3* paralogs in fish shows strong homology to mammalian *RLN3*, where they are predominantly expressed in the periaqueductal grey, a region homologous to NI in mammals. Additionally, it is known that *rln3a* is expressed in a broader range of tissues (including gonad) than *rln3b*, indicating that *rln3a* and *rln3b* exhibit spatial (and perhaps temporal) subfunctionalization [8, 9, 14]. Our expression analyses indicate both coexpression of the *rln3* paralogs with *rxfp3-1* and *rxfp3-2* genes, and also possible subfunctionalization of the receptor since all of the *rxfp3-1* and *rxfp3-2* (and even *rxfp3-3*) genes are highly expressed in brain, while *rxfp3-2a* and *rxfp3-2b* are additionally expressed in the ovary, but at lower levels, mimicking the expression pattern of its candidate ligand, *rln3a*.

### 3.2. Receptors for the Insl5 Paralogs in Teleosts Are Difficult to Resolve

Resolving the ligand-receptor pairings for the Insl5-Rxfp4 system in teleosts is more difficult. We hypothesized that the Rxfp3-3 and Rxfp3-4 descendents (Figure 1) are the potential receptors for Insl5a and Insl5b (Figure 2, supplementary Figure S3). Specifically, we hypothesized that, in teleosts, Rxfp3-3a1, Rxfp3-3a2, and Rxfp3-3b are candidate receptors for Insl5a while Rxfp3-4 (aka Rxfp4) is the receptor for Insl5b; in zebrafish, the loss of *rxfp3-4* was compensated by the gain of *rxfp3-3a3* (Figure 2), and the latter could serve as the receptor for Insl5b (Figures 3 and 4). Despite this prediction, the selection and expression data provided little evidence for which receptors may bind to the two teleost *insl5* paralogs (Figure 9). The selection profile of teleost *rxfp4* is the best match for that of both *insl5a* and *insl5b*, but all three *rxfp3-3*-type receptors are dominated by purifying selection and have selection profiles similar to those of *rln3*. On the other hand, the experimental data in zebrafish (which lacks *rxfp4*) indicate that *insl5a* is expressed in intestine and gonads and *insl5b* is expressed predominantly in intestine, and both paralogs exhibit low but significant expression in brain. This is consistent with the pattern in mammals, but the only receptor expressed at high levels in intestine was *rxfp3-3b*. The failure to find stronger evidence of coexpression of additional receptors for the Insl5 paralogs may be caused, in part, by the endocrine action of Insl5 and its expression in peripheral tissues [18, 24], many of which were not examined here, or possibly by developmental regulation of one or both of the *insl5* paralogs. Three of the other Rxfp3-3 receptor genes, *rxfp3-3a1*, *rxfp3-3a2*, and *rxfp3-3a3*, were all additionally expressed in brain and male gonads, therefore if Insl5a is a ligand for these receptors, teleosts may have expanded and subfunctionalized the role of the Insl5 peptides involved in the HPG axis. Further experimental work, including *in situ* hybridization, should be performed on *insl5* and *rxfp3-3* receptors in teleosts to thoroughly assess this hypothesis. Furthermore, the coexpression of *insl5*- and *rxfp3/4*-type genes in a teleost species other than zebrafish should be performed since zebrafish possesses a slightly unique suite of genes (Table S2), which did not allow for qPCR analyses of *rxfp4*.

### 3.3. The INSL3-RXFP2 System Exhibits Similar Expression Patterns in Mammals and Zebrafish

While teleosts exhibit a clear expansion of the *rln/insl* and *rxfp* genes involved in neuroendocrine pathways, the 3R duplicates of *rln* and *insl3* and their corresponding *rxfp1/2*-type receptors expanded minimally. We find good support for the hypothesis that Insl3-Rxfp2 are ligand-receptor pairs in teleosts: their selection profiles are highly similar and, in zebrafish, which contain two *rxfp2* paralogs (*rxfp2a* and *rxfp2b*), both receptor genes are highly expressed in gonads, although *rxfp2b* is additionally quite highly expressed in brain. Previously, it was shown that *insl3* expression in zebrafish shows strong parallels to that in mammals: *in situ* and qPCR analyses on male gonads reveal that it is expressed predominantly in Leydig cells [8], and the more thorough qPCR analyses presented here further demonstrate that it is very abundantly expressed in male gonads, but also in female ovaries. Current *in situ* analysis (underway in our laboratory) has also revealed the specificity of *rxfp2a* and *rxfp2b* expression in Leydig cells (unpublished data). On the other hand, although *rxfp2-like* (which among teleosts is only present in zebrafish) has a similar selection profile to *insl3*, we found it to be predominantly expressed in brain, rendering interpretation difficulty, and we favor the hypothesis that Rxfp2-like is an alternate receptor for Rln (see Figures 4 and 9). 

### 3.4. RLN-RXFP1 System in Placental Mammals and Teleosts: Conserved Receptor but Rapidly Evolving Ligand in Mammals

The only ligand-receptor pair for which there was a poor correlation in the nature of selection was RLN-RXFP1 in mammals. While *RXFP1 *genes in mammals and teleosts have evolved in similar ways, the gene coding for the hormone relaxin, *rln,* has been subject to purifying and neutral evolution in teleosts, but has been the target of strong positive selection in mammals (see Figure 5(c), Table S3). In accordance with two recent studies showing the strong role of selection on the relaxin locus [25, 26], we find that approximately 50% of the codons in mammalian *RLN* show evidence of positive selection, whereas no sites in teleost *rln *do. Additionally, the qPCR expression pattern of *rxfp1* in zebrafish shows broad but low levels of expression across multiple tissues, including gonad and brain. Using RT-PCR and *in situ* analyses in zebrafish, Donizetti et al. [27] showed that expression of *rxfp1* in zebrafish brain begins early in development and shows strong overlap with that of *RXFP1* in humans. Based on the similar amino acid sequence of Rln and Rln3 in teleosts, they propose that Rxfp1 could be an additional receptor for Rln3a and/or Rln3b in teleosts. A study comparing the expression of *rln3a, rln3b*, and *rln* in eel using *in situ* and qPCR analyses [9] found that the expression of teleost *rln *is similar to that of *rln3*, but with lower expression in brain and higher in gonads, similar to that observed in which expression was predominantly found in gonad. This pattern is supported by our hypothesis for the evolution of the system in which the ancestral ligand molecule is hypothesized to have functioned in both reproductive and neuroendocrine pathways (Figure 3).

### 3.5. Evidence for Differential Selection in Teleost Versus Mammalian rln/insl-rxfp Genes Suggests Functional Divergence of the Ligand-Receptor Coding Sequences

Although we have focused on the similarities in the evolution of mammalian and teleost *RLN*/*INSL*-*RXFP* genes, the analysis of codon-specific positive selection revealed that mammalian and teleost genes have been subject to differential selection and that some receptor domains are the targets of more selection than others. For this analysis, sites were deemed to be subject to codon-specific selection if, when comparing a particular branch of the phylogenetic tree, there was evidence that certain amino acids were selected to be different from those in the “background” lineage for the same gene. By analyzing the genes in this way, we found that for the *RXFP1/2*-type genes, the LDLa-LRR region generally showed high levels of selection, not surprisingly, since they are involved in receptor-ligand signaling [5]. Functional studies have shown that the LRR region is important for the binding of the cognate ligand; the LDLa module is essential for cAMP accumulation which takes place after the ligand is recognized and bound [5]. Apart from these regions, the only other two regions which were identified as having more than 20% of the sites subject to selection for *RXFP1/2* genes were ICL3 and ECL2. 

In general, lineage-specific selection was higher for the *RXFP3/4-*type genes: all domains were found to have more than 20% of the amino acids subject to positive selection except for four regions of the transmembrane domain (TM1, TM2, TM3, andTM7) and ECL1. Of particular interest is the fact that for the *RXFP3/4*-type genes, ICL1 is equally important as ICL3 in terms of selection. The finding that ICL3 (both receptor types) and ICL1 (RXFP3/4-type receptors) are targets of selection suggests that a major component of selection for the RXFP receptors concerns downstream receptor signaling rather than selection for ligand binding *per se*. 

## 4. Conclusions

Although the majority of the relaxin family genes originated prior to the divergence of osteichthyans, the fate of the family in teleosts and mammals is markedly different owing to the differential retention and diversification of genes in each lineage. Earlier studies suggested that teleosts only possessed *relaxin 3-* and *rxfp3-*like genes and proposed that RLN and INSL3 were neurohormones that recruited their RXFP1/2-type receptors after the divergence of mammals [28], a view that is inconsistent with the data presented here and elsewhere [2, 8, 29, 30]. The goal of this study was to establish a theoretical background for further experimental work on the *rln/insl-rxpf* systems in teleosts. Although the study was limited because its methodology relied on the known ligand-receptor pairings and expression data from mammals as a reference, our analyses suggest that the orthologs of the four 2R-derived ligand genes (*RLN*, *INSL3*, *RLN3*, and *INSL5*) have similar ligand-receptor pairings in teleosts and mammals (with the exception of the unusual situation with Rln-Rxfp1). Despite these similar patterns, there is also evidence of differential selection on specific amino acids in mammalian versus teleost lineages, suggesting functional divergence in the two lineages. 

It is interesting that the RLN/INSL peptides diversified their reproductive functions in mammals, owing to local duplications at the relaxin locus [23, 25, 26, 29], while teleosts underwent a massive diversification of the genes believed to be involved in neuroendocrine regulation (*rln3*/*insl5*-*rxpf3/4*). Overall, we find evidence that many of these “additional” receptor genes in teleosts have characteristics of the RLN3-RXFP3 system, that is, slow evolution and predominant expression in the brain, while the primary receptors for the two Insl5 paralogs in teleosts remain obscure. Nevertheless, we find that teleosts greatly expanded and probably subfunctionalized the role of the rxfp3-2- and rxfp3-3-derived receptors; their cognate ligands and their physiological functions should be the focus of future experimental work.

## 5. Materials and Methods

### 5.1. Selection Profiles of Candidate Ligand-Receptor Pairs

We obtained sequences and performed an alignment based on the coding sequence for the RLN/INSL-RXFP genes from 5 teleosts (zebrafish, medaka, fugu, tetraodon, and stickleback) and 11 placental mammals (human, rhesus, cow, pig, horse, dog, guinea pig, mouse, rat, rabbit, and elephant) as described previously [2]. The accession numbers of all genes are listed in Tables S4 and S7 in Yegorov and Good [2], and the alignment is available upon request.

We calculated the proportion of codons in ligand and receptor pairs estimated to be subject to purifying, neutral, or positive selection using the sites model in PAML [22]. Next, to assess whether teleost ligand or receptor genes have been subject to adaptive divergent selection, we used several methods that examine the ratio of nonsynonymous to synonymous (*d*_*N*_/*d*_*S*_) substitutions. Because *d*_*S*_ provides an approximation of the neutral rate of substitution, *ω* = *d*_*N*_/*d*_*S*_ ratios are used to determine selection pressure on genes or codon positions, with *ω* > 1 indicative of positive Darwinian selection [31].

**Site Models**

We employed models that allow *ω* to vary among sites and tested a series of models to look for evidence of positive selection. First, we compared model M7 (beta) versus M8 (beta + *ω*) to test for evidence of positive selection and then compared model 8 versus model 8a to assess whether the evidence for positive selection was actually caused by a relaxation of purifying selection (or true positive selection); for both comparisons we used the site model tests in PAML [32]. Likelihood ratio tests (LRTs) were constructed to compare model M7 versus M8 and M8a versus M8. Twice the log likelihood difference between models was compared with a chi-square distribution with number of degrees of freedom (*df*) calculated as the difference in the number of estimated parameters between models. Model M8 was additionally used to identify codon sites under positive selection using a Bayes Empirical Bayes (BEB) criterion.

**Branch-Site Models**

We hypothesized that at least some of the receptor genes may have experienced lineage-specific positive selection in mammals versus teleosts. To examine this we used the branch-site model A of Zhang et al. [22], which tests whether the members of a user-defined clade (branch) on a phylogenetic tree exhibit evidence of codon-specific selection relative to the remaining (background) lineages. Tests of positive selection were made by comparing the branch-site model A in which (*d*_*N*_/*d*_*S*_) > 1 (alternative hypothesis) to the model A in which *d*_*N*_/*d*_*S*_ = 1 fixed (null hypothesis) and by setting the foreground branch to the base of the clade containing the relaxin family ortholog in teleosts and the background to the same ortholog in mammals or tetrapods (depending on the tree structure) or vice versa. Analysis of the branch-site model A was done using CODEML from the PAML package (PAML v. 4.2); models were compared using the likelihood ratio test with 1 degree of freedom and, where significant, the posterior probability that a codon was under positive selection was estimated using the Bayes Empirical Bayes (BEB) procedure [22].

### 5.2. Quantitative Expression Analysis in Zebrafish Tissues Animals

Sexually mature male and female zebrafish (*Danio rerio*) from the Tübingen AB strain were used. Animal housing [33] and experimentation were consistent with Dutch national regulations and were approved by the Utrecht University Animal Use and Care Committee.

**RNA Isolation and cDNA Synthesis**

Various tissues (heart, intestine, testis, ovary, muscle, gill, brain, and eye) were dissected from male and female adult zebrafish and immediately flash frozen in liquid nitrogen.Tissue samples from 3 individual zebrafish, for each gender, were combined for each replicate and the RNA was isolated using the FastRNA Pro Green kit (Bio 101 Systems), according to the manufacturer's recommendations. Three independent RNA isolations (biological replicates), each containing pooled tissues from 3 individual fish, were performed for each tissue per sex. Possible genomic DNA contamination was removed from each total RNA fraction with the RNAse-free DNase Treatment & Removal kit (Ambion), which includes a final step to remove the DNAse I from the reaction. Next, cDNA synthesis was performed with 2 *μ*g of each total RNA samples, as described previously [34].

**Real-Time, Quantitative PCR**

Primers (Table S6) for real-time, quantitative PCR (qPCR) to detect zebrafish *rln/insl* and *rxfp* mRNAs were designed and validated for specificity and amplification efficiency on serial dilutions of testis cDNA [35] using SYBR Green-based assays (Applied Biosystems, Foster City, CA, USA). All primers were designed on different exons, except for the primers detecting the *rxfp3* cDNAs, since all *rxfp3* genes are single-exon genes. Moreover, each qPCR run was followed by a melt curve analyses to exclude potential PCR amplifications from genomic DNA contamination. To normalize the data, a TaqMan Gene Expression Assay was acquired to detect the endogenous control RNA, eukaryotic *18S ribosomal* RNA (Applied Biosystems). To examine the relative expression of genes across tissues, the relative fold change of the genes of interest was normalized to the *18S ribosomal RNA* reference gene and to a calibrator (calculated as the mean expression of all genes) (supplementary Figures S2 (ligands) and S3 (receptors). All qPCRs and calculations (using the ΔΔC_T_ method) were performed as described previously [35–37]. To compare the expression levels of all relaxin family peptide and receptor genes in *whole *zebrafish tissues, expression levels were additionally corrected for the total RNA yield per tissue per sex (Figure 8).

## Supplementary Material

Table A1. Expression studies describing expression of relaxin family ligands and their receptors in mammals and teleosts.
Table A2: Summary of the orthologous/paralogous relationships of the genes coding for relaxin family peptides and their receptors in humans, the gnathostome ancestor (post 2R ancestor), zebrafish and the remaining teleosts for which whole genome sequencing data is available.
Table A3. Results of the site model of codon specific selection in mammalian and teleost RLN/INSL genes.
Table A4. Results of the site model of codon specific selection in mammalian and teleost RXFP genes.
Table A5. Results of the analyses using the branch-site model A of Zhang et al. [30] on relaxin family orthologues, specifying either teleosts or mammals as the foreground branch on which the alternate (alt) hypothesis of positive selection will be compared to the null model (*ω*=1, fixed).
Table A6. Primers used to determine the relative expression of rln/insl and rxfp genes in zebrafish.
Figure A1. Histograms presenting the proportion of sites showing evidence of positive selection in the branch-site model comparing teleost versus mammalian gene.
Figure A2. Relative expression of relaxin ligand genes in zebrafish tissues.
Figure A3. Relative expression of relaxin receptor genes in zebrafish tissues.

## Appendix

See Supplementary Tables S1, S2, S3, S4, S5, and S6 and Figures S1, S2, and S3 (see Supplementary materials available online at doi:10.1155/2012/310278).
