# Supplementary material for: New Insights into Ligand-Receptor Pairing and Coevolution of Relaxin Family Peptides and Their Receptors in Teleosts
Source: Int J Evol Biol. 2012 Sep 13;2012:310278. doi: 10.1155/2012/310278 (PMC3449138; doi:10.1155/2012/310278)
Supplement: Supplementary file 1 — Table A1. Expression studies describing expression of relaxin family ligands and their receptors in mammals and teleosts. Table A2: Summary of the orthologous/paralogous relationships of the genes coding for relaxin family peptides and their receptors in humans, the gnathostome ancestor (post 2R ancestor), zebrafish and the remaining teleosts for which whole genome sequencing data is available. Table A3. Results of the site model of codon specific selection in mammalian and teleost RLN/INSL genes. Table A4. Results of the site model of codon specific selection in mammalian and teleost RXFP genes. Table A5. Results of the analyses using the branch-site model A of Zhang et al. [30] on relaxin family orthologues, specifying either teleosts or mammals as the foreground branch on which the alternate (alt) hypothesis of positive selection will be compared to the null model (ω=1, fixed). Table A6. Primers used to determine the relative expression of rln/insl and rxfp genes in zebrafish. Figure A1. Histograms presenting the proportion of sites showing evidence of positive selection in the branch-site model comparing teleost versus mammalian gene. Figure A2. Relative expression of relaxin ligand genes in zebrafish tissues. Figure A3. Relative expression of relaxin receptor genes in zebrafish tissues. [file 310278.f1.docx]

APPENDIX

New insights into ligand-receptor pairing and co-evolution of relaxin family peptides and their receptors in teleosts

*International Journal of Evolutionary Biology*

**Authors:** *Sara Good, Sergey Yegorov, Joran Martijn, Jens Franck, Jan Bogerd*

Table A1. Expression studies describing expression of relaxin family ligands and their receptors in mammals and teleosts.

| **Study** | **Methods** | **Organism, tissue** | **Findings** |
| --- | --- | --- | --- |
| Adam et al (1993): INSL3 | Northern blot  In situ | Boar testis cDNA library | They find INSL3 only expressed in testis |
| Balvers et al., (1998) insl3 | RT-PCR, in situ | Mouse ovary and testis | Expressed in adult male mouse testis and in ovarian luteal cells during cycle, pregnancy and lactation but lower levels |
| Bathgate et al (1996) insl3 | RT-PCR, cDNA library screening, in situ | Cow, ovary and testis | Argue that because cow have lost RLN, INSL3 may be highly expressed in thecal cells to replaces role of RLN |
| Kawamura et al (2004) insl3, rxfp2 | northern | Rat | Find that LH stimulates INSL3 in females, regulating oocyte maturation |
| Tanaka et al (2005): rln3 | immunohistochemistry | Rat brain | Find RLN3 predominantly expressed in *nucleus incertus*. |
| Bathgate et al. (2002): rln3 | RT-PCR northerns | mouse | Highest in brain, but also in spleen thymus, ovary |
| Hudson et al (1984) | northern | human | H2 expressed in ovary |
| Gunnerson et al (1995): rln | RT-PCR, RNase protection assay, immunohistochemistry | Rat, multiple tissues | Brain, uterus, prostrate gland, kidney, pancreas |
| Bathgate et al (2002): rln | RT-PCR, northern | Corpus luteum, tammar wallaby | Expression of RLN unaffected by pregnancy state of females |
| Osheroff and Ho (1993): rln | Northern, in situ | Rat brain and heart | Find RLN in rat male and female brain, receptors more widely distributed; find receptors also in heart but not ligand |
| [Hossain, et al., 2008](#_ENREF_1) insl5 |  | Human | proposed to be involved in gut contractility |
| Conklin et al (1999): insl5 | Northern, qPCR | Human and mouse | Human: rectal, colon, uterus. Mouse same + thymus + testis |
| Dun et al (2006) insl5 | RT-PCR | Mouse brain | Find expression in hypothalamus and pituitary, neuroendocrine |
| Liu et al (2005): insl5, RXFP4 | qPCR | human | INSL5 : fetal brain, kidney, lung, ovary, thymus, thyroid, placenta, pituitary ; RXFP4 : leukocytes, colon, low in placenta and other tissues |
| Hsu et al (2002) RXFP1 and RXFP2 |  |  | Wide and divergent expression of both receptors show roles in brain, reproduction, renal, cardiovascascular and other functions |
| Anand-Ivell et al (2006)  RXFP2 | RT-PCR, immunohistochemistry  Ligand-binding assays | Rat testicular tissue, leydig cells, epididymis | Suggest rxfp2 expression independent of HPG pathway, expressed in leydig, gubernaculum, and epididymis (no known function) |
| Boels and Schaller (2003) RXFP4 | Northern, tissue array | Human, multiple tissues | Expressed in many peripheral tissues including heart, liver, spleen, ovary, but even small amounts in brain |

Table A1 References

Adam IM, Burkhardt E, Benahmed M, et al. (1993) Cloning of a cDNA for a novel insulin-like peptide of the testicular Leydig cells. J Biol Chem 268: 26668-26672.

Akhter Hossain M, Bathgate RAD, Kong CK, Shabanpoor F, Zhang S, et al. (2008) Synthesis, Conformation, and Activity of Human Insulin-Like Peptide 5 (INSL5). ChemBioChem 9: 1816-1822.

Anand-Ivell RJK, Relan V, Balvers M, Coiffec-Dorval I, Fritsch M, et al. (2006) Expression of the Insulin-Like Peptide 3 (INSL3) Hormone-Receptor (LGR8) System in the Testis. Biology of Reproduction 74: 945-953.

Balvers M, Spiess A-N, Domagalski R, Hunt N, Kilic E, et al. (1998) Relaxin-Like Factor Expression as a Marker of Differentiation in the Mouse Testis and Ovary. Endocrinology 139: 2960-2970.

Bathgate R, Balvers M, Hunt N, Ivell R (1996) Relaxin-like factor gene is highly expressed in the bovine ovary of the cycle and pregnancy: sequence and messenger ribonucleic acid analysis. Biology of Reproduction 55: 1452-1457.

Bathgate RA; Samuel CS; Burazin TC; Layfield S; Claasz AA; Reytomas IG; Dawson NF; Zhao C; Bond C; Summers RJ; Parry LJ; Wade JD; Tregear GW (2005). Human relaxin gene 3 (H3) and the equivalent mouserelaxin (M3) gene. Novel members of the relaxinpeptide family. J. Biol. Chem. 277 (2) 1148-57.

Boels K, Schaller HC. Identification and characterisation of GPR100 as a novel human G-protein-coupled bradykinin receptor. Br J Pharmacol. 2003;140:932–938.

Conklin D, Lofton-Day CE, Haldeman BA, Ching A, Whitmore TE, Lok S, Jaspers S (Sep 1999). "Identification of INSL5, a new member of the insulin superfamily". *Genomics* **60** (1): 50–6

Dun SL, Brailoiu E, Wang Y, Brailoiu GC, Liu-Chen L-Y, et al. (2006) Insulin-Like Peptide 5: Expression in the Mouse Brain and Mobilization of Calcium. Endocrinology 147: 3243-3248.

Gunnerson, J.M., Crawford, R.J. and Tregear, G.W. (1995) Expression of the relaxin gene in rat tissues. Mol. Cell. Endocrinol., 110, 55–64.

Hudson P, John M, Crawford R, Haralambidis J, Scanlon D, Gorman J, Tregear G, Shine J, Niall H. Relaxin gene expression in human ovaries and the predicted structure of a human preprorelaxin by analysis of cDNA clones. *EMBO J.*1984 Oct;**3**(10):2333–2339.

Hsu SY, Nakabayashi K, Nishi S, Kumagai J, Kudo M, et al. (2002) Activation of Orphan Receptors by the Hormone Relaxin. Science 295: 671-674.

Kawamura K, Kumagai J, Sudo S, Chun SY, Pisarska M, et al. (2004) Paracrine regulation of mammalian oocyte maturation and male germ cell survival. Proc Natl Acad Sci U S A 101: 7323-7328.

Liu C, Kuei C, Sutton S, Chen J, Bonaventure P, et al. (2005) INSL5 is a high affinity specific agonist for GPCR142 (GPR100). J Biol Chem 280: 292-300.

Osheroff PL, Ho WH (1993) Expression of relaxin mRNA and relaxin receptors in postnatal and adult rat brains and hearts. Localization and developmental patterns. Journal of Biological Chemistry 268: 15193-15199.

Tanaka M, Iijima N, Miyamoto Y, Fukusumi S, Itoh Y, et al. (2005) Neurons expressing relaxin 3/INSL 7 in the nucleus incertus respond to stress. European Journal of Neuroscience 21: 1659-1670.

**Table A2:** Summary of the orthologous/paralogous relationships of the genes coding for relaxin family peptides and their receptors in humans, the gnathostome ancestor (post 2R ancestor), zebrafish and the remaining teleosts for which whole genome sequencing data is available. Whether genes originated via WGD (2R, 3R) or small scale duplications (SSD’s) is indicated. Data following Yegorov and Good, 2012. The RLN locus in mammals underwent successive SSD, but this occurred after 2R and will not be covered here. *† =* pseudogene.

| Human Ortholog | Post-2R name | Teleost post-3R genes excl. zebrafish | fish specific SSD | Zebrafish genes |
| --- | --- | --- | --- | --- |
| *RLN2* | *Rln* | *rln* |  | *rln* |
| *INSL3* | *insl3* | *insl3* |  | *insl3* |
| *RLN3* | *rln3* | *rln3a* |  | *rln3a* |
|  |  | *rln3b* |  | *rln3b* |
| *INSL5* | *insl5* | *insl5a* |  | *insl5a* |
|  |  | *insl5b* |  | *insl5b* |
| *RXFP1* | *rxfp1* | *rxfp1* |  | *rxfp* |
| *RXFP2* | *rxfp2* | *rxfp2* |  | *rxfp2a* |
|  |  | *rxfp2b†* |  | *rxfp2b* |
|  | *rxfp2-like* | *†* |  | *rxfp2-like* |
| *RXFP3* | *rxfp3-1* | *rxfp3-1* |  | *rxfp3-1* |
| *†* | *rxfp3-2* | *rxfp3-2a* |  | *rxfp3-2a* |
|  |  | *rxfp3-2b* |  | *rxfp3-2b* |
| *†* | *rxfp3-3* | *rxfp3-3a* | *rxfp3a1, rxfp3a2* | *rxfp3-3a1, rxfp3-3a2, rxfp3-3a3* SSD |
|  |  | *rxfp3-3b* |  | *rxfp3-3b* |
| *RXFP4* | *rxfp3-4* | *rxfp4* |  | *†* |
|  |  |  |  |  |

**Table A3.** Results of the site model of codon specific selection in mammalian and teleost RLN/INSL genes. Model 7 test for evidence of purifying selection, model 8, for positive selection and model 8a, tests whether there has been a relaxation of purifying selection. Models are compared using a likelihood ratio test (LRT), which is chi-square distributed with the degrees of freedom equal to the difference in the number of parameters between models. Sites identified as being subject to positive selection (i.e. when model 8 is significantly better than both models 7 and 8a) are selected based on Bayes Empirical Bayes (BEB) critiera. p<0.0001=***, p<0.001=**, p<0.01=*, p<0.05=+. The null and alternative models are significantly different when LRT > 3.841

| **Model** | **L** | **LRT** | **positively selected sites** |
| --- | --- | --- | --- |
|  |  |  |  |
| **insl5mam** |  |  |  |
| **model7** | -1171.19 |  |  |
| **model8** | -1159.49 | 23.40** | **36M**,5.54; 37S***, 5.743; 38 R***,5.749** |
| **model8a** | -1166.07 | 13.15** |  |
| **insl5fish** |  |  |  |
| **model7** | -1115.8 |  |  |
| **model8** | -1115.83 | -7.6E^-05^ |  |
| **model 8a** | -1115.83 | 0.0072 |  |
| **rln – mammals** |  |  |  |
| **model 7** | -1977.52 |  |  |
| **model 8** | -1973.10 | 8.853** | **1T**,1.47;5K**,1.43;16L,**,1.47;42Y**,1.45;43I**,1.40;44K**,1.45;51N**,1.49;52V**,1.47;2D*,1.41;4K^+^,1.49;8A^+^,1.34;17Q^+^,1.38;23S*,1.47;30W*,1.40;32G^+^,1.34;47D^+^,1.34;64R*,1.47** |
| **model 8a** | -1975.75 | 3.54 |  |
| **rln-fish** |  |  |  |
| **model7** | -409.43 |  |  |
| **model8** | -409.43 | -0.0009 |  |
| **model8a** | -409.41 | -0.044 |  |
| **insl3mam** |  |  |  |
| **model 7** | -894.14 |  |  |
| **model8** | -894.14 | -0.0001 |  |
| **mode8a** | -894.12 | -0.034 |  |
| **insl3 fish** |  |  |  |
| **model 7** | -659.22 |  |  |
| **model8** | -654.10 | 10.24 | **36I***,23.28;37 R***,23.28** |
| **model8a** | **-655.93** | **3.660** |  |

**Table A4.** Results of the site model of codon specific selection in mammalian and teleost RXFP genes. model 7 test for evidence of purifying selection, model 8, for positive selection and model 8a, tests whether there has been a relaxation of purifying selection. Models are compared using a likelihood ratio test (LRT), which is chi-square distributed, with degrees of freedom equal to the difference in the number of parameters between models. Sites identified as being subject to positive selection (i.e. when model 8 is significantly better than both models 7 and 8a) are selected based on Bayes Empirical Bayes (BEB) criterion, p<0.0001=***, p<0.001=**, p<0.01=*, p<0.05=+. The null and alternative models are significantly different when LRT > 3.841

| **Model** | **L** | **LRT** | **positively selected sites** |
| --- | --- | --- | --- |
| **rxfp1mam** |  |  |  |
| **model 7** | -10047.7 |  |  |
| **model 8** | -10045.0 | 5.386 | **108A,**,1.61;57Y^+^,1.40;76V^+^,1.39;79 LV,1.39** |
| **model 8a** | -10045.74 | 1.30 |  |
| **rxfp1fish** |  |  |  |
| **model7** | -6385.64 |  |  |
| **model8** | -6384.36 | 2.550 | **62^+^,1.28** |
| **model8a** | -6384.40 | 0.064 |  |
| **rxfp2mam** |  |  |  |
| **model7** | -9852.03 |  |  |
| **model 8** | -9849.52 | 5.013 | **99 M*,1.45;282 D^+^,1.35, 610 S**,1.46** |
| **model 8a** | -9850.16 | 1.27 |  |
| **rxfp2fish** |  |  |  |
| **model7** | -5658.65 |  |  |
| **model8** | -5655.15 | 6.99 | **62F*,1.46;90A^+^,1.36;338L**, 1.48,339K*,1.45** |
| **model8a** | -5658.08 | 5.86 |  |
| **rxxfp3-1mam** |  |  |  |
| **model7** | -3639.82 |  |  |
| **model8** | -3636.17 | 7.31 | **241K**,1.97** |
| **model8a** | -3637.87 | 3.40 |  |
| **rxfp3-1fish** |  |  |  |
| **model7** | -2544.38 |  |  |
| **model8** | -2544.38 | -0.001 |  |
| **model8a** | -2544.34 | -0.083 |  |
| **rxfp3-2fish** |  |  |  |
| **model7** | -4116.60 |  |  |
| **model8** | -4116.60 | -0.099 |  |
| **model8a** | -4116.55 | -0.099 |  |
| **rxfp3-3fish** |  |  |  |
| **model7** | -7151.16 |  |  |
| **model8** | -7151.16 | -0.004 |  |
| **model8a** | -7150.91 | -0.497 |  |
| **rxfp4fish** |  |  |  |
| **model7** | -2557.24 |  |  |
| **model8** | -2552.94 | 8.597 | **169K**,3.37;83V^+^,3.10,122S^+^,3.07** |
| **model 8a** | -2553.25 | 0.632 |  |
| **rxfp4mam** |  |  |  |
| **model7** | -3598.04 |  |  |
| **model8** | -3594.45 | 7.167 |  |
| **model8a** | **-3594.46** | **0.016** |  |

**Table A5.** Results of the analyses using the branch-site model A of Zhang *et al*. [30] on relaxin family orthologues, specifying either teleosts or mammals as the foreground branch on which the alternate (alt) hypothesis of positive selection will be compared to the null model (ω=1, fixed). The proportion of sites subject to purifying (p0), nearly neutral (p1) and positive selection (p2) and the estimate of ω (ω_2_) in the free model are given as is the 2 Δ Likelihood (L) of the model, and the codon positions (using Humans (for mammals) or *T. nigroviridis*as (for teleosts) as the reference sequence) of the sites estimated to be subject to positive selection. The null and alternative models are significantly different when 2 Δ L > 3.841. p<0.0001=***, p<0.001=**, p<0.01=*,

| **Gene** | **Model** | **Foreground**  **branch** | **Parameter** | **Δ dF** | **2 Δ L** | **Positively selected sites** |
| --- | --- | --- | --- | --- | --- | --- |
| *RXFP1* | A  (alt) | mammals *RXFP1* | p0=.79, p1=.11,  p2=.084, ω_2_=0.07 | 1 | 7.8 | 41T*, 154 N*, 229G*, 337 R*, 507I* 525N*, 574 S*, 577 T*, 629 F*, 662 N*, 688 L* (+ 18 sites with BEB >0.5, <0.9) |
|  | A  (alt) | teleosts  *rxfp1* | p0=.82, p1=.12,  p2=.06, ω_2_=.08 | 1 | 5.1 | 42 S*, 198F*, 265S*, 415 T*, 177 I*, 297*, (+24 sites with BEB >0.5, <0.9) |
| *RXFP2* | A  (alt) | Mammals *RXFP2* | p0=.75, p1=.15,  p2=0.1, ω_2_=0.1 |  | 10.3 | 41T*, 162*, 241G*, 246Y*, 322M*, 352 R**, 544 N*, 595S*, 598 ***, 53 F*, 717 L ** (+27 sites with BEB >0.5, <0.9) |
|  | A  (alt) | Teleosts  *Rxfp2* | p0=.43, p1=.56,  p2=0.0, ω_2_ | 3 | 2.9 | (5 sites with BEB >0.5, <0.9) |
| *RXFP3* | A  (alt) | Mammals *RXFP3* | p0=.91, p1=.036,  p2=.06, ω_2_=.05 | 3 | 4.9 | 77K*, 156A*, 169V*, 170K*, 207S*, + (7 sites with BEB >0.5, <0.9) |
|  | A  (alt) | Teleosts  *Rxfp3* | p0=.93, p1=.05,  p2=.02, ω_2_=0.06 | 3 | 3.1 | 5 sites selected with BEB >0.5, <0.9 |
| *RXFP4* | A  (alt) | Mammals *RXFP4* | p0=0.80, p1=0.07 p2=.12,  ω_2_=0.076 | 3 | 6.7 | 83A**, 182 L*, 189S* , 285 P*, 292 T* +(18 sites with BEB >0.5, <0.9) |
|  | A  (alt) | Teleosts  *Rxfp4* | p0=0.76,p1=0.13 p2=0.11  ω_2_=0.085 |  | 7.1 | 39R***, 219A*, 235R*, *235, **239**, + (14 sites with BEB >0.5, <0.9) |

**Table A6**. Primers used to determine the relative expression of rln/insl and rxfp genes in zebrafish.

| Gene | Forward primer | Reverse primer |
| --- | --- | --- |
| *Rln* | 5’-CATCCGGGCGGTGATCTT-3’ | 5’-CCACCGAGAAGTTCCTCTTCCT-3’ |
| *rln3a* | 5’-ATCCCGATGGAAACGCTCTT-3’ | 5’-GCGGCATTACTGTCATATGAGTTG-3’ |
| *rln3b* | 5’-CGCTGGAGGAGATCTCTGGAT-3’ | 5’-CAGAGGCCTCGTCATCATGAG-3’ |
| *Insl3* | 5’-TCGCATCGTGTGGGAGTTT-3’ | 5’-TGCACAACGAGGTCTCTATCCA-3’ |
| *insl5a* | 5’-GAAGTGCAGGCGGATGTCA-3’ | 5’-GACCCCTCCATTCAGAAAACCT-3’ |
| *insl5b* | 5’-GAGGCGGGTCCAAACTGAA-3’ | 5’-CTCTTCTTTCTCGGTCCATTTCTG-3’ |
| *Rxfp1* | 5’-GGAGGTCGAGATCCCTGGAA-3’ | 5’-GCTGTTGATGGGCAGAATGAA-3’ |
| *rxfp2-like* | 5’-GGAGAAACCTGGTGCTAGATGCTAT-3’ | 5’-CACAAAAGCCAGCAGATTCAGA-3’ |
| *rxfp2a* | 5’-CAATTCCAGTCTCTGTCAGCACAT-3’ | 5’-CTCAACGTCATTCTCCGCAAA-3’ |
| *rxfp2b* | 5’-CTGCCAGACTCTGTGCCCATA-3’ | 5’-AGTCGTGATGCTATTACCCTCGAA-3’ |
| *rxfp3-1* | 5’-GTTTTGACGCTTCCCTTTTGG-3’ | 5’-AAAAACACGCTGGCGTACATG-3’ |
| *rxfp3-2a* | 5’-AAATCGTTTGGATGCGTAAAGC-3’ | 5’-GCGCATCGCTCTCATATAAAGC-3’ |
| *rxfp3-2b* | 5’-CTACATTCACGCTACCGGCATAA-3’ | 5’-CTGTTAGAGCCAAACCCATCACA-3’ |
| *rxfp3-3a1* | 5’-GGAGACGCCATGTGCAAGAT-3’ | 5’-CATCGCCGTCAGGAAGAAGA-3’ |
| *rxfp3-3a2* | 5’-AAAGAAGTCTGTGTCTGTGAAGTGGAT-3’ | 5’-GTCACAGTGGAGAAAATGGAAGTTG-3’ |
| *rxfp3-3a3* | 5’-CGCAATAGGGTTAATCGGGAAT-3’ | 5’-GCTCTGCCTGGAGTGTTTCACT-3’ |
| *rxfp3-3b* | 5’-GCCGGCGGAGCATGA-3’ | 5’-ACGGATTTGGTGACTCTGGATCT-3’ |

**A)**

**B)**

**C)**

**D)**

**Figure A1.** Histograms presenting the proportion of sites showing evidence of positive selection in the branch-site model comparing teleost versus mammalian gene. A) For mammalian Rxfp1, teleosts show more evidence of lineage specific positive selection than mammals, although the regions of selection differ between the two lineages- in mammals, the first four regions of the (Ldla-LRR2) and ICL3 have a high proportion of sites subject to positive selection, while for teleosts regions LRR2-LRR9, ICL2 and ECL1 exhibit strong evidence of positive selection. B) For Rxfp2, mammals exhibit the strongest selection in regions LRR6 and ICL3, while teleosts exhibit the highest level of selection for ECL1. C) For Rxfp3, mammals show evidence of positive selection for ICL3, and ECL1, while teleosts show little evidence of selection. D) Lastly, for Rxfp4, mammals again show evidence on intra-cellular loops, ICL1 and ICL3, while teleosts show evidence of selection primarily at ECL1 and ECL3. Collectively this suggests greater differentiation in intracellular signaling in mammals and in extracellular signaling in teleosts. LDLa – low density lipoportin module A, LRR- leucine rich repeat, TM – transmembrane domain, ICL – intracellular loop, ECL extracellular loop

**Figure A2.** Relative expression of relaxin ligand genes in zebrafish tissues. Per graph, the expression of a gene relative to the average expression of that gene in 2 μg RNA of all tissues in both sexes is shown. Three biological replicates were used to determine the relative expression

**Figure A3.** Relative expression of relaxin receptor genes in zebrafish tissues. Per graph, the expression of a gene relative to the average expression of that gene in 2 μg RNA of all tissues in both sexes is shown. Three biological replicates were used to determine the relative expression
